# Treatment of Class III Malocclusion and Anterior Crossbite with Aligners: A Case Report

**DOI:** 10.3390/medicina58050603

**Published:** 2022-04-27

**Authors:** Alessio Danilo Inchingolo, Assunta Patano, Giovanni Coloccia, Sabino Ceci, Angelo Michele Inchingolo, Grazia Marinelli, Giuseppina Malcangi, Chiara Di Pede, Mariagrazia Garibaldi, Anna Maria Ciocia, Antonio Mancini, Giulia Palmieri, Biagio Rapone, Fabio Piras, Filippo Cardarelli, Ludovica Nucci, Ioana Roxana Bordea, Antonio Scarano, Felice Lorusso, Delia Giovanniello, Stefania Costa, Gianluca Martino Tartaglia, Daniela Di Venere, Gianna Dipalma, Francesco Inchingolo

**Affiliations:** 1 Department of Interdisciplinary Medicine, University of Bari “Aldo Moro”, 70124 Bari, Italy; ad.inchingolo@libero.it (A.D.I.); assuntapatano@gmail.com (A.P.); giovanni.coloccia@gmail.com (G.C.); s.ceci@studenti.uniba.it (S.C.); angeloinchingolo@gmail.com (A.M.I.); graziamarinelli@live.it (G.M.); giuseppinamalcangi@libero.it (G.M.); c.dipede1@studenti.uniba.it (C.D.P.); mgr.garibaldi@libero.it (M.G.); anna.ciocia1@gmail.com (A.M.C.); dr.antonio.mancini@gmail.com (A.M.); giuliapalmieri13@gmail.com (G.P.); biagiorapone79@gmail.com (B.R.); dott.fabio.piras@gmail.com (F.P.); drfilippocardarelli@libero.it (F.C.); daniela.divenere@uniba.it (D.D.V.); giannadipalma@tiscali.it (G.D.); francesco.inchingolo@uniba.it (F.I.); 2 Multidisciplinary Department of Medical-Surgical and Dental Specialties, University of Campania “Luigi Vanvitelli”, Via L. De Crecchio 6, 80138 Naples, Italy; ludovica.nucci@unicampania.it; 3 Department of Oral Rehabilitation, Faculty of Dentistry, Iuliu Hațieganu University of Medicine and Pharmacy, 400012 Cluj-Napoca, Romania; 4 Department of Innovative Technologies in Medicine and Dentistry, University of Chieti-Pescara, 66100 Chieti, Italy; ascarano@unich.it; 5 Department of Thoracic Surgery, Hospital “San Camillo Forlanini”, 00152 Rome, Italy; giovanniellodelia@gmail.com; 6 Department of Biomedical and Dental Sciences and Morphofunctional Imaging, Section of Orthodontics, School of Dentistry, University of Messina, 98125 Messina, Italy; stefaniacosta94@gmail.com; 7 Department of Biomedical, Surgical and Dental Sciences, School of Dentistry, University of Milan, 20100 Milan, Italy; gianluca.tartaglia@unimi.it; 8 Fondazione IRCCS Cà Granda, Ospedale Maggiore Policlinico, 20100 Milan, Italy

**Keywords:** orthodontics, corrective orthodontics, invisalign, removable orthodontic appliances, tooth movement techniques

## Abstract

The article describes the orthodontically treated case of a 25-year-old patient with skeletal and dental class III malocclusion, anterior crossbite, which caused functional and aesthetic problems, occlusal trauma, and incisor wear. Treatment with transparent aligners was proposed to meet the patient’s needs, using the sequential distalization protocol. While sequential distalization is well documented for class II malocclusion treatment in maxillary arch teeth, further investigations are necessary for class III malocclusions. In fact, lower teeth movements are more complex due to mandibular bone density and the presence of the third molars, which are often extracted to perform distalization. In addition, the use of intermaxillary elastics helps control the proclination of the anterior teeth as a reaction to distalizing forces. At the end of the treatment, the patient reached molar and canine class I and positive overjet and overbite. The inclination of lower incisors and the interincisal angle have improved, resulting in aesthetic and functional enhancement.

## 1. Introduction

Class III malocclusion is a maxillofacial disorder characterized by a concave profile due to mandibular protrusion, maxillary retrusion or a combination of both. There is a significant amount of scientific literature on the rapidly growing orthopaedic approach to class III malocclusions, which have a strong genetic influence on their aetiology [1,2,3,4,5,6,7].

In adult patients, it is essential to distinguish between the basal skeletal class III, for which surgical treatment is elective, and functional class III, or pseudo-class III, in which, due to a discrepancy between maximal intercuspation and centric occlusion, and therefore an occlusal interference, the patient in maximum intercuspation advances the mandible. In these cases, occlusal improvement can be achieved with orthodontic therapy alone [8,9,10,11,12,13]. Although the combination of orthognathic surgery and orthodontic therapy is considered the gold standard for class III malocclusion, if the patient decides not to undergo surgery, orthodontic treatment alone may help compensate for mild skeletal class III malocclusion. In this way, both the function and aesthetics of the patient can be improved [14,15].

This paper reports the case of a patient with skeletal and dental class III malocclusion, negative overjet and overbite who did not accept a combination of orthognathic surgery and orthodontic therapy. The patient was treated with clear aligners to achieve dental compensation by applying the sequential distalization protocol of 50% of the lower arch teeth. At the end of the treatment, an improvement of the overbite, overjet and class III molar was achieved with retroclination of the lower incisors to compensate skeletal malocclusion.

Recently, an increasing number of adult patients have requested orthodontic treatment, desiring an effective and comfortable alternative to fixed multibracket therapy [16]. Transparent aligners can meet this demand because, with proper planning and patient cooperation, the effectiveness of this therapy is comparable to fixed orthodontic treatment [17,18,19,20].

## 2. Case Report

**Figure 1:**
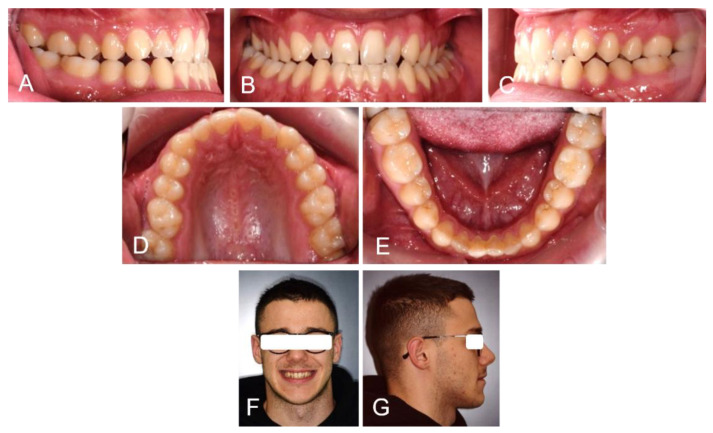
Extra and intraoral photos of the patient before treatment: Right lateral occlusion (**A**); Frontal occlusion (**B**); Left lateral occlusion (**C**); Upper occlusion (**D**); Lower occlusion (**E**); Patient smile (**F**) and right profile (**G**).

A 25-year-old patient had skeletal and dental class III malocclusion, anterior crossbite and incisal head-to-head relationship. This produced not only functional and aesthetic alterations but also occlusal trauma and incisor wearing. During childhood, the patient had already been orthodontically treated with multibrackets equipment and a palatal expander. Unfortunately, an orthodontic relapse occurred during adulthood, so the patient asked for re-treatment with a lower aesthetic impact and greater comfort than his previous experience (Figure 1).

**Figure 2:**
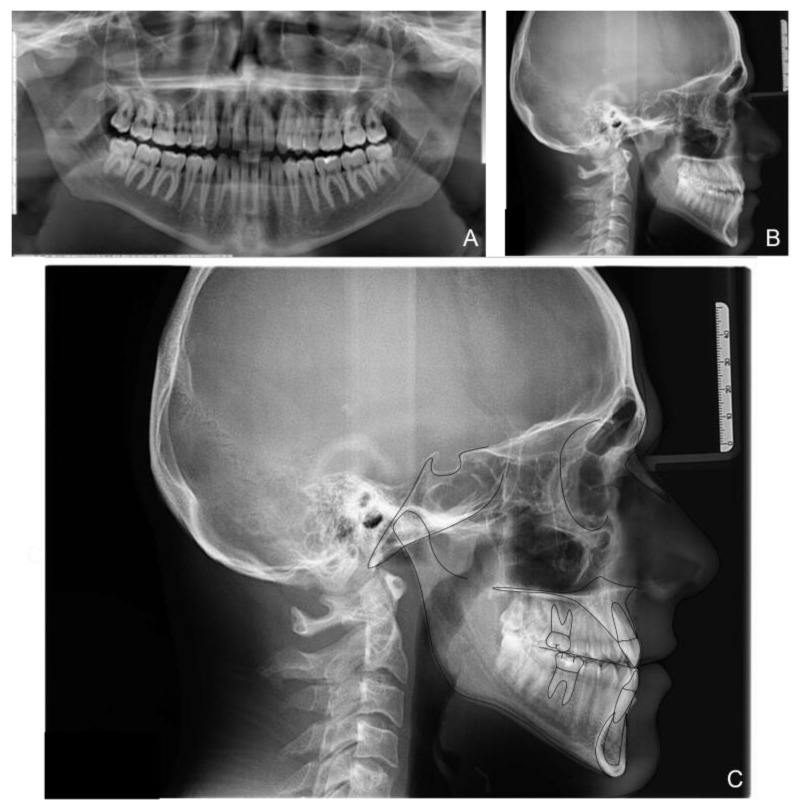
Patient X-rays, cephalometry tracing and analysis before treatment: panoramic X-ray (**A**); latero-lateral X-ray of the skull before treatment (**B**); Cephalometric tracing (**C**).

The orthopanoramic X-ray showed the physiological presence of all teeth and their good condition. The latero-lateral skull X-ray revealed a class III hyperdivergent skeletal malocclusion, a slight overbite and a negative overjet. Clinical examination also indicated minor teeth crowding with Angle class III malocclusion and head-to-head incisors relationship (Figure 2 and Table 1).

Intraoral scans were carried out, and models were analysed, highlighting a Bolton discrepancy of 0.30 mm in the lower arch due to the lower incisors’ greater mesiodistal dimension. Before orthodontic treatment, extractions of the lower two-third molars were carried out to create a distal space for the second molars.

**Figure 3:**
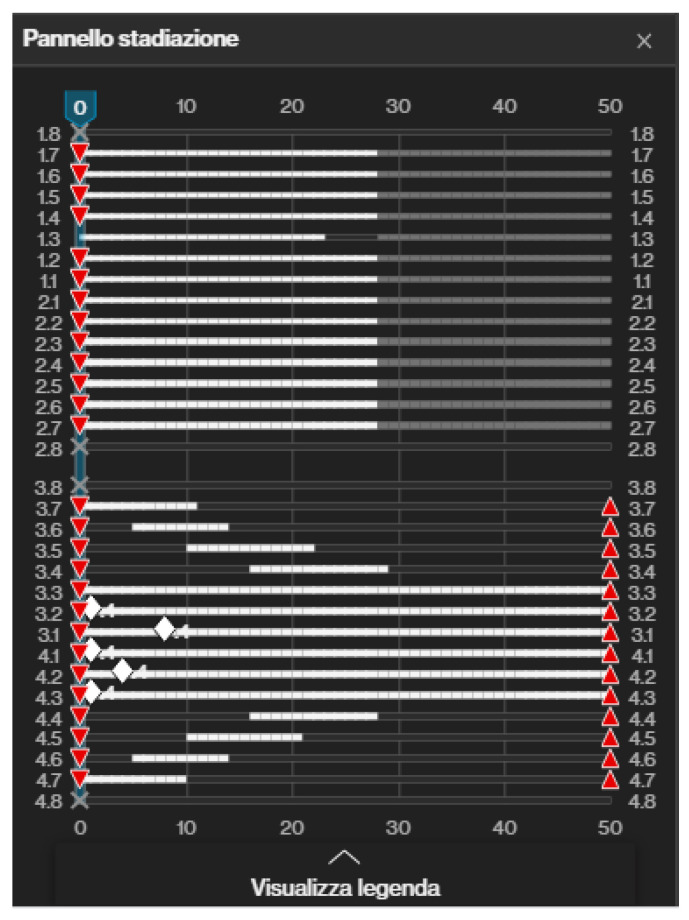
Invisalign^®^ (Align Technology, Tempe, AZ, USA) software staging table where it is possible to appreciate the “ladder” scheme of distalization. In the upper arch all teeth were moved at the same time. In the lower arch, lower teeth were distalized in a sequential way, while anterior teeth were moved from the beginning. Red triangles mean that all teeth had attachments.

The orthodontic setup included the sequential staging of movements (Figure 3); the second molars were distalized first, and after 50% of their movement, the movement of the first molars began. The planned distalization was 2 mm on the right side and 1 mm on the left side. Once the distalization of the second molar was completed, the distalization of the second premolar began. In the same pattern, all the lower elements were distalized. A slight expansion of the upper and lower arches was necessary to allow the crowding to resolve. In addition, mild proclination of the upper anterior teeth was planned to create spaces distal to the upper lateral incisors.

Although the lower incisors were in the correct cephalometric position, it was necessary to retroclinate them and perform an interproximal reduction (IPR) to resolve the anterior overjet. IPR was indicated because the incisors were triangular in shape [21].

**Figure 4:**
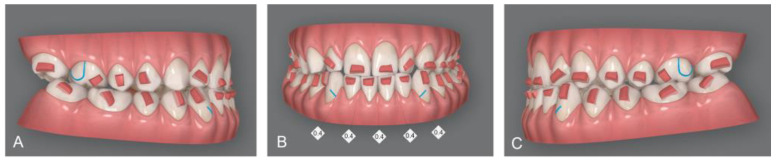
Invisalign^®^ software display of attachments, programmed IPR and cuts for elastic: Right lateral vision of virtual setup (**A**); Frontal vision of virtual setup; IPR of 0.4 mm from canine to canine was planned (**B**); Left lateral vision of virtual setup (**C**).

Upon delivery of the first transparent aligners, rectangular retention attachments were applied to the posterior teeth and rectangular attachments with gingival bevels on the lower anterior teeth to ensure maximum retention of the aligners. In addition, posterior attachments provided the proper retention and control of molars and premolars distal tipping, while retention attachments on lower canines and upper first molars prevented side effect movements with the application of class III intermaxillary elastics. Interproximal reduction (IPR) between lower incisors of 2 mm was planned (Figure 4). After third molar extraction, composite attachments and metal buttons for elastic support were bonded. Orthodontic elastics plus clear aligners were delivered to the patient [22].

**Figure 5:**
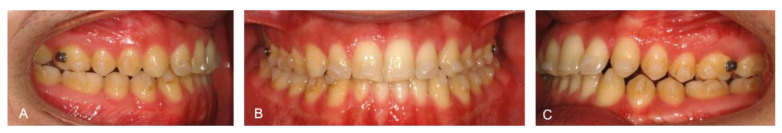
Intra-oral pictures of the patient after the first treatment phase: Right lateral occlusion (**A**); Frontal occlusion (**B**); Left lateral occlusion (**C**).

**Figure 6:**
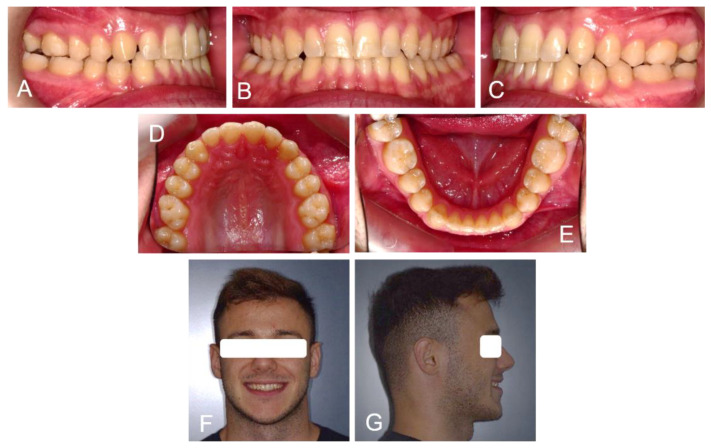
Intra and extra-oral pictures of the patient at the end of treatment: Right lateral occlusion (**A**); Frontal occlusion (**B**); Left lateral occlusion (**C**); Upper arch (**D**); Lower arch (**E**); Patients smile (**F**) and profile (**G**).

**Figure 7:**
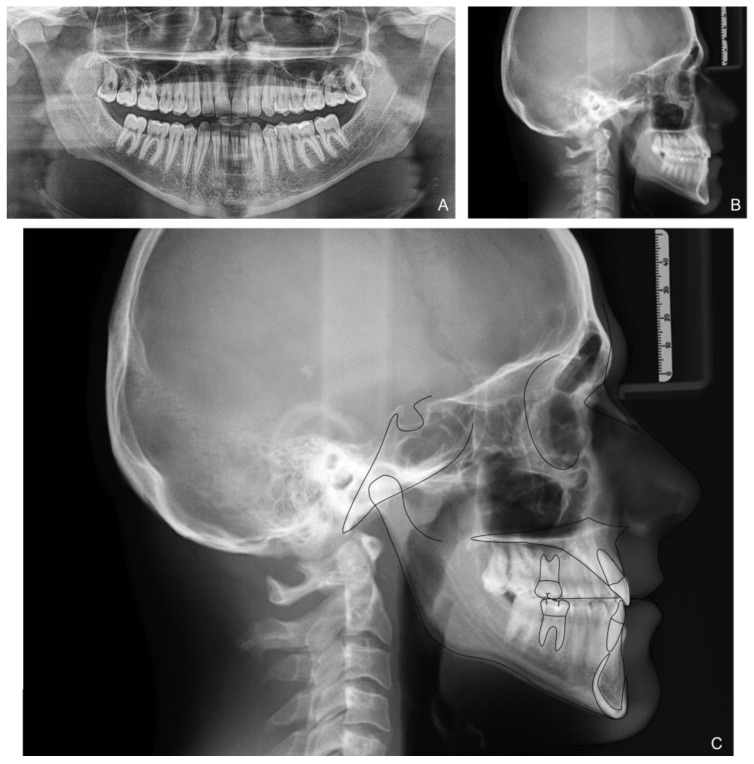
Patient X-rays and cephalometry after treatment: Orthopanoramic X-ray (**A**); Latero-lateral skull X-ray (**B**); Cephalometric tracing (**C**).

**Figure 8:**
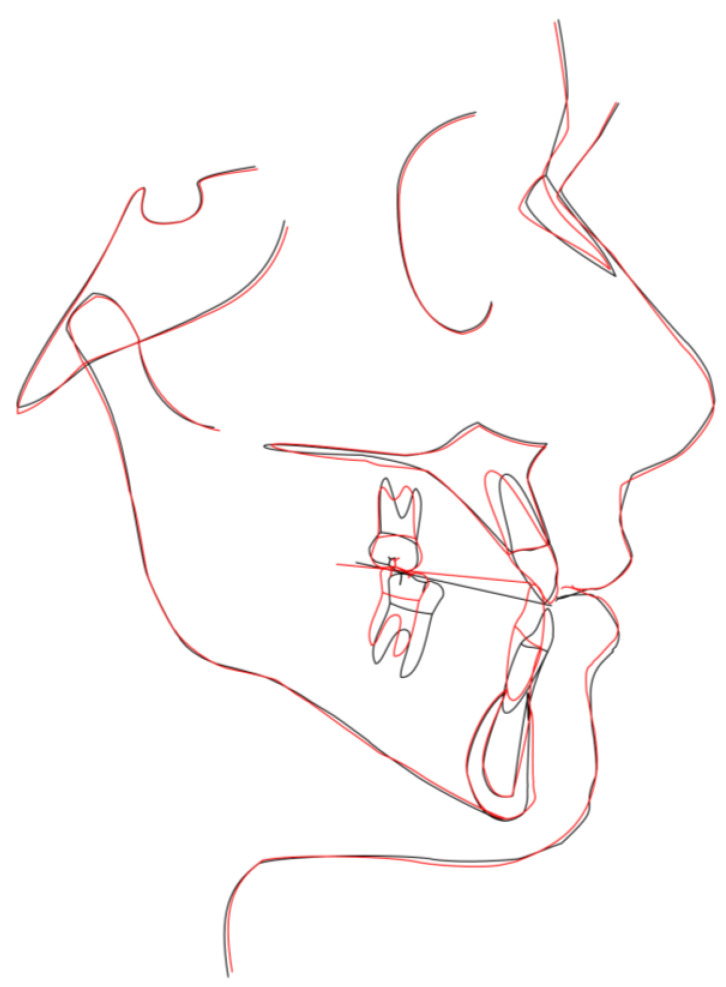
Cephalometric tracing superimposition: red line t1 and black line t0.

The patient correctly wore the aligners for 22 h a day with class III elastics anchored on the upper first molars and lower canines. After 12 months of treatment, the patient had improved tooth class, a positive anterior overjet, resolution of the posterior crossbite, and improved upper and lower alignment. At the end of the first phase of treatment (Figure 5), slight refinement was required. A few months later, the patient consolidated the molar and canine class I relationship, achieved a positive overjet and overbite, and resolved midline misalignment (Figure 6). At the end of the refinement phase, the patient was required to wear a long-term upper and lower Essix retention device during the night. Due to the transverse discrepancy between the upper and lower arch caused by class III malocclusion, to maintain adequate periodontal health of the upper posterior sectors, it was preferred not to expand the upper arch excessively and therefore not to resolve the crossbite of the second molars. From a cephalometric point of view (Figure 7 and Table 2), the inclination of the lower incisors and interincisal angle improved, which resulted in an aesthetic and functional enhancement. Pre-post-cephalometric evaluation, made with Deltadent^®^ software (Outside format, Pandino, Italy), is reported in Table 3 and cephalometric tracing superimposition is shown in Figure 8.

## 3. Discussion

Treatment with clear aligners allows better sagittal control in bite malocclusions because it allows more significant arch opening and the application of intermaxillary elastics to control the loss of anterior anchorage due to distalizing forces [17,23,24,25,26].

Orthodontic treatment with aligners is increasingly sought after by patients of all ages due to aesthetic and comfort requirements, but orthodontists are also increasingly adopting it. The digital workflow allows for a 3D scan of the dental arches and the design of tooth movements. It is possible to choose which teeth to move individually, as if using a segmented orthodontic system, or to move all the teeth simultaneously, as if using a straight wire or functional orthodontic system [27,28,29,30,31,32,33].

Furthermore, the versatility of the aligner system is another aspect to consider, among the advantages they offer. In particular, it is possible to design a sequential distalization of the upper or lower jaw to solve a dental class II or III, respectively, in a segmented manner [34,35,36].

The disadvantages are related to the need for maximum collaboration. The patient must wear the aligners for at least 22 h a day [37,38,39]. Additionally, their biomechanics are more complex than fixed therapy [27,40,41,42].

Sequential distalization involves initially moving the most distal teeth while keeping the rest of the arch still anchored. Subsequently, the mesial teeth are immediately moved, and the whole arch is shifted distally [17,43,44]. The correction of the altered molar class takes a long time and is visible after many months of treatment. For this reason, to maintain high levels of cooperation, it is necessary to solve the problem of anterior crowding from the first aligner. Many case reports have demonstrated the possibility of achieving molar sequential distalization in patients with the Invisalign^®^ system [45,46,47,48].

Simon et al. demonstrated the high predictability of molar distalization with aligners by planning attachments and movements of 2.6 mm. Aligners can prevent distal tipping and molar extrusion as a reaction force to distalization [49,50,51].

Kamy Malekian et al. reported two cases: 2.5 and 3 mm distalization, measured from the distal surface of the lower molar to Rickett’s vertical line in the cephalometric analysis. To achieve this result, it was necessary to extract the third molars. Furthermore, considering that the correction occurs by tooth movement, there is a greater demand for anchoring control. Indeed, a loss of anchorage could occur due to the mutual reaction force [17,52,53].

The treatment of dental class II with sequential distalization is well recorded and widely used by orthodontists. In contrast, a more in-depth study is required to lower teeth distalization for class III malocclusions due to the greater bone density and frequent dysodontiasis of the III lower molars [54,55,56]. In both cases, it is necessary to wear intermaxillary elastics (with a class II or III force vector) together with the aligners to control the proclination of the anterior teeth. The sequential distalization can be observed graphically in the “phases” table, which shows to which aligner a given tooth movement corresponds. Therefore, it is possible to visualise a kind of “ladder” shape in the sequential movement diagram, which shows how a single tooth moves for the first aligners. Therefore, during the later stages, a maximum of two teeth will move simultaneously, and at the end of the movement of each tooth, its position will be blocked. Even if the teeth do not move during the distalization movement, it is necessary to reinforce anchorage with intermaxillary elastics. The Invisalign^®^ design software can insert cuts into the aligners themselves to allow direct application of the elastic or adhesive buttons for direct application of the elastic on a tooth [18,57,58,59,60,61].

The patient in this study did not accept orthodontic treatment associated with orthognathic surgery but accepted a compromise of orthodontic treatment with lower molar distalization, retroclination of lower incisors and proclination of upper incisors to improve overjet and overbite and correct class III. The patient preferred to wear orthodontic aligners instead of fixed multibracket appliances. Aligners can be more aesthetic than fixed orthodontics and allow patients to clean their teeth more easily [36,62]. Like a fixed appliance, aligners allowed the application of class III elastics to reinforce lower anterior anchorage during lower first and second molars distalization after the extraction of the lower third molars. It should be emphasised that the patient was very cooperative, wearing aligners and intermaxillary elastics. The advantage of the aligners was that every movement was programmed before starting the treatment, and the movements were planned with sequential staging. In fact, only the lower second molars were moved during the first phases of the treatment. All the other teeth were blocked, and no movement was planned. After moving 50% of the second molars, the first molars started to move. These movements are called “sequential”.

Long-term stability will be monitored in subsequent years. It is mandatory to control orthodontic relapse with removable retention, considering that this treatment was a compromise with compensation of skeletal class III, which is easy to relapse.

## 4. Conclusions

The patient in this case report saw aesthetic and comfort needs achieved with clear aligners. In addition, the patient’s excellent compliance allowed good results to be achieved without damaging the patient’s periodontium. It can be said that the sequential distalization protocol of the lower teeth, which is less studied than the sequential distalization of the upper teeth, leads to good compromise results while avoiding, as in this case, orthognathic surgery. The patient refused the latter option when possible treatment alternatives were presented.

## Figures and Tables

**Table 1:** Cephalometric values before treatment.

Cephalometric Parameters	Min	Mean	Max	t0
**Sagittal Skeletal Relations**				
Maxillary Position	78.5°	82°	85.5°	82.5°
Mandibular Position	76.5°	80°	83.5°	84.4°
Sagittal Jaw Relation	−0.5°	2°	4.5°	−1.9°
**Vertical Skeletal Relations**				
Maxillary Inclination	5°	8°	11°	4.3°
Mandibular Inclination	30.5°	33°	35.5°	31°
Vertical Jaw Relation	19°	25°	31°	27.6°
**Dento Basal Relations**				
Maxillary Incisor Inclination	104°	110°	116°	106.9°
Mandibular Incisor Inclination	87°	94°	101°	81.4°
Mandibular Incisor Compensation	0	2	4	3.7
**Dental Relations**				
Overjet	1	3.5	6	−0.9
Overbite	−0.5	2	4.5	−0.9
Interincisal Angle	126°	132°	138°	140.6°

**Table 2:** Final cephalometric values.

Cephalometric Parameters	Min	Mean	Max	t1
**Sagittal Skeletal Relations**				
Maxillary Position	78.5°	82°	85.5°	82.5°
Mandibular Position	76.5°	80°	83.5°	84.4°
Sagittal Jaw Relation	−0.5°	2°	4.5°	−1.9°
**Vertical Skeletal Relations**				
Maxillary Inclination	5°	8°	11°	4.3°
Mandibular Inclination	30.5°	33°	35.5°	31°
Vertical Jaw Relation	19°	25°	31°	26.7°
**Dento Basal Relations**				
Maxillary Incisor Inclination	104°	110°	116°	115.2°
Mandibular Incisor Inclination	87°	94°	101°	84.9°
Mandibular Incisor Compensation	0	2	4	1.9
**Dental Relations**				
Overjet	1	3.5	6	3.5
Overbite	−0.5	2	4.5	3.4
Interincisal Angle	126°	132°	138°	145°

**Table 3:** Comparison of pre- (t0) and post- (t1) cephalometric values.

Cephalometric Parameters	Min	Mean	Max	t0	t1
**Sagittal Skeletal Relations**					
Maxillary Position	78.5°	82°	85.5°	82.5°	82.5°
Mandibular Position	76.5°	80°	83.5°	84.4°	84.4°
Sagittal Jaw Relation	−0.5°	2°	4.5°	−1.9°	−1.9°
**Vertical Skeletal Relations**					
Maxillary Inclination	5°	8°	11°	4.3°	4.3°
Mandibular Inclination	30.5°	33°	35.5°	31°	31°
Vertical Jaw Relation	19°	25°	31°	27.6°	26.7°
**Dento Basal Relations**					
Maxillary Incisor Inclination	104°	110°	116°	106.9°	115.2°
Mandibular Incisor Inclination	87°	94°	101°	81.4°	84.9°
Mandibular Incisor Compensation	0	2	4	3.7	1.9
**Dental Relations**					
Overjet	1	3.5	6	−0.9	3.5
Overbite	−0.5	2	4.5	−0.9	3.4
Interincisal Angle	126°	132°	138°	140.6°	145°

## Data Availability

All experimental data to support the findings of this study are available by contacting the corresponding author.

## References

[1] Dehesa-Santos A., Iber-Diaz P., Iglesias-Linares A. (2021). Genetic Factors Contributing to Skeletal Class III Malocclusion: A Systematic Review and Meta-Analysis. Clin. Oral Investig..

[2] Jiménez-Silva A., Carnevali-Arellano R., Vivanco-Coke S., Tobar-Reyes J., Araya-Díaz P., Palomino-Montenegro H. (2021). Craniofacial Growth Predictors for Class II and III Malocclusions: A Systematic Review. Clin. Exp. Dent. Res..

[3] Rongo R., D’Antò V., Bucci R., Polito I., Martina R., Michelotti A. (2017). Skeletal and Dental Effects of Class III Orthopaedic Treatment: A Systematic Review and Meta-Analysis. J. Oral Rehabil..

[4] Inchingolo A.D., Patano A., Coloccia G., Ceci S., Inchingolo A.M., Marinelli G., Malcangi G., Montenegro V., Laudadio C., Palmieri G. (2021). Genetic Pattern, Orthodontic and Surgical Management of Multiple Supplementary Impacted Teeth in a Rare, Cleidocranial Dysplasia Patient: A Case Report. Medicina.

[5] Ngan P. (2005). Early Timely Treatment of Class III Malocclusion. Semin. Orthod..

[6] Nucci L., Costanzo C., Carfora M., d’Apuzzo F., Franchi L., Perillo L. (2021). Dentoskeletal Effects of Early Class III Treatment Protocol Based on Timing of Intervention in Children. Prog. Orthod..

[7] Maspero C., Abate A., Inchingolo F., Dolci C., Cagetti M.G., Tartaglia G.M. (2022). Incidental Finding in Pre-Orthodontic Treatment Radiographs of an Aural Foreign Body: A Case Report. Children.

[8] Alfaifi A.H. (2021). Restorative Management and Treatment of Pseudo-Class III Malocclusion. Case Rep. Dent..

[9] Kale B., Buyukcavus M.H. (2020). Comparison of Three-Dimensional Soft-Tissue Evaluations between Skeletal and Pseudo-Class III Malocclusions. Sci. Rep..

[10] Patianna A.G., Ballini A., Meneghello M., Cantore S., Inchingolo A.M., Dipalma G., Inchingolo A.D., Inchingolo F., Malcangi G., Lucchese A. (2019). Comparison of Conventional Orthognathic Surgery and “Surgery-First” Protocol: A New Weapon against Time. J. Biol. Regul. Homeost. Agents.

[11] Adina S., Dipalma G., Bordea I.R., Lucaciu O., Feurdean C., Inchingolo A.D., Septimiu R., Malcangi G., Cantore S., Martin D. (2020). Orthopedic Joint Stability Influences Growth and Maxillary Development: Clinical Aspects. J. Biol. Regul. Homeost. Agents.

[12] Cardarelli F., Patano A., Montenegro V., Malcangi G., Coloccia G., Inchingolo A.D., Marinelli G., Laudadio C., Dipalma G., Di Venere D. (2021). Elastodontic therapy un nuovo approccio alla terapia ortodontica funzionale. Il Dent. Mod..

[13] Fabozzi F.F., Nucci L., Correra A., d’Apuzzo F., Franchi L., Perillo L. (2021). Comparison of Two Protocols for Early Treatment of Dentoskeletal Class III Malocclusion: Modified SEC III versus RME/FM. Orthod. Craniofac. Res..

[14] Mora Martínez M.A., Pesqueira Melgarejo R., Hernández Espinosa G., De Silva Dávila J.L., Rodríguez Chávez J.A. (2015). Alteración dentofacial clase III tratado con camuflaje: Reporte de caso clínico. Rev. Mex. Ortod..

[15] Farronato M., Maspero C., Abate A., Grippaudo C., Connelly S.T., Tartaglia G.M. (2020). 3D Cephalometry on Reduced FOV CBCT: Skeletal Class Assessment through AF-BF on Frankfurt Plane—Validity and Reliability through Comparison with 2D Measurements. Eur. Radiol..

[16] Montinaro F., Nucci L., Carfora M., d’Apuzzo F., Franchi L., Perillo L. (2021). Modified SEC III Protocol: Vertical Control Related to Patients’ Compliance with the Chincup. Eur. J. Orthod..

[17] Malekian K., Parrini S., Garino F., Deregibus A., Castroflorio T. (2019). Mandibular Molar Distalization with Clear Aligners in Class III Patients. J. Aligner Orthod..

[18] Papadimitriou A., Mousoulea S., Gkantidis N., Kloukos D. (2018). Clinical Effectiveness of Invisalign^®^ Orthodontic Treatment: A Systematic Review. Prog. Orthod..

[19] Laudadio C., Inchingolo A.D., Malcangi G., Limongelli L., Marinelli G., Coloccia G., Montenegro V., Patano A., Inchingolo F., Bordea I.R. (2021). Management of Anterior Open-Bite in the Deciduous, Mixed and Permanent Dentition Stage: A Descriptive Review. J. Biol. Regul. Homeost. Agents.

[20] Di Venere D., Corsalini M., Nardi G.M., Laforgia A., Grassi F.R., Rapone B., Pettini F. (2017). Obstructive Site Localization in Patients with Obstructive Sleep Apnea Syndrome: A Comparison between Otolaryngologic Data and Cephalometric Values. Oral Implantol..

[21] De Felice M.E., Nucci L., Fiori A., Flores-Mir C., Perillo L., Grassia V. (2020). Accuracy of Interproximal Enamel Reduction during Clear Aligner Treatment. Prog. Orthod..

[22] Robertson L., Lee D., Eimar H., El-Bialy T. (2019). Treatment of a Challenging Class III Malocclusion Case Using Invisalign Clear Aligners and Micro-Osteoperforation: A Case Report. J. Aligner Orthod..

[23] Rossini G., Parrini S., Castroflorio T., Deregibus A., Debernardi C.L. (2014). Efficacy of Clear Aligners in Controlling Orthodontic Tooth Movement: A Systematic Review. Angle Orthod..

[24] Lombardo L., Colonna A., Carlucci A., Oliverio T., Siciliani G. (2018). Class II Subdivision Correction with Clear Aligners Using Intermaxilary Elastics. Prog. Orthod..

[25] Caruso S., Nota A., Ehsani S., Maddalone E., Ojima K., Tecco S. (2019). Impact of Molar Teeth Distalization with Clear Aligners on Occlusal Vertical Dimension: A Retrospective Study. BMC Oral Health.

[26] Wheeler T.T. (2017). Orthodontic Clear Aligner Treatment. Semin. Orthod..

[27] Jindal P., Juneja M., Siena F.L., Bajaj D., Breedon P. (2019). Mechanical and Geometric Properties of Thermoformed and 3D Printed Clear Dental Aligners. Am. J. Orthod. Dentofac. Orthop..

[28] Marinelli G., Inchingolo A.D., Inchingolo A.M., Malcangi G., Limongelli L., Montenegro V., Coloccia G., Laudadio C., Patano A., Inchingolo F. (2021). White Spot Lesions in Orthodontics: Prevention and Treatment. A Descriptive Review. J. Biol. Regul. Homeost. Agents.

[29] Patano A., Cirulli N., Beretta M., Plantamura P., Inchingolo A.D., Inchingolo A.M., Bordea I.R., Malcangi G., Marinelli G., Scarano A. (2021). Education Technology in Orthodontics and Paediatric Dentistry during the COVID-19 Pandemic: A Systematic Review. Int. J. Environ. Res. Public Health.

[30] Inchingolo A.D., Patano A., Coloccia G., Ceci S., Inchingolo A.M., Marinelli G., Malcangi G., Montenegro V., Laudadio C., Pede C.D. (2022). The Efficacy of a New AMCOP^®^ Elastodontic Protocol for Orthodontic Interceptive Treatment: A Case Series and Literature Overview. Int. J. Environ. Res. Public Health.

[31] Farronato M., Farronato D., Inchingolo F., Grassi L., Lanteri V., Maspero C. (2021). Evaluation of Dental Surface after De-Bonding Orthodontic Bracket Bonded with a Novel Fluorescent Composite: In Vitro Comparative Study. Appl. Sci..

[32] Cantore S., Ballini A., Farronato D., Malcangi G., Dipalma G., Assandri F., Garagiola U., Inchingolo F., De Vito D., Cirulli N. (2016). Evaluation of an Oral Appliance in Patients with Mild to Moderate Obstructive Sleep Apnea Syndrome Intolerant to Continuous Positive Airway Pressure Use: Preliminary Results. Int. J. Immunopathol. Pharmacol..

[33] Brugnami F., Meuli S., Caiazzo A., Marrocco S., Scopelliti D. (2021). Three-Dimensional Digital Planning of Class III Decompensation with Clear Aligners: Hard and Soft Tissue Augmentation with Concomitant Corticotomy to Stretch the Limits of Safe Orthodontic Treatment. J. Oral Biol. Craniofacial Res..

[34] Mehta S., Patel D., Yadav S. (2021). Staging Orthodontic Aligners for Complex Orthodontic Tooth Movement. Turk. J. Orthod..

[35] Edelmann A., English J.D., Chen S.J., Kasper F.K. (2020). Analysis of the Thickness of 3-Dimensional-Printed Orthodontic Aligners. Am. J. Orthod. Dentofac. Orthop..

[36] Cozzani M., Sadri D., Nucci L., Jamilian P., Pirhadirad A.P., Jamilian A. (2020). The Effect of Alexander, Gianelly, Roth, and MBT Bracket Systems on Anterior Retraction: A 3-Dimensional Finite Element Study. Clin. Oral Investig..

[37] Sahm G., Bartsch A., Witt E. (1990). Reliability of Patient Reports on Compliance. Eur. J. Orthod..

[38] Montenegro V., Inchingolo A.D., Malcangi G., Limongelli L., Marinelli G., Coloccia G., Laudadio C., Patano A., Inchingolo F., Bordea I.R. (2021). Compliance of Children with Removable Functional Appliance with Microchip Integrated during COVID-19 Pandemic: A Systematic Review. J. Biol. Regul. Homeost. Agents.

[39] Di Venere D. (2017). Correlation between Parodontal Indexes and Orthodontic Retainers: Prospective Study in a Group of 16 Patients. Oral Implantol..

[40] Putrino A., Barbato E., Galluccio G. (2021). Clear Aligners: Between Evolution and Efficiency-A Scoping Review. Int. J. Environ. Res. Public Health.

[41] Ballini A., Cantore S., Scacco S., Perillo L., Scarano A., Aityan S.K., Contaldo M., Cd Nguyen K., Santacroce L., Syed J. (2019). A Comparative Study on Different Stemness Gene Expression between Dental Pulp Stem Cells vs. Dental Bud Stem Cells. Eur. Rev. Med. Pharmacol. Sci..

[42] Kravitz N.D., Moshiri M., Nicozisis J., Miller S. (2020). Mechanical Considerations for Deep-Bite Correction with Aligners. Semin. Orthod..

[43] Marra P., Nucci L., Jamilian A., Perillo L., Itro A., Grassia V. (2020). Odontoma in a Young and Anxious Patient Associated with Unerupted Permanent Mandibular Cuspid: A Case Report. J. Int. Oral Health.

[44] Di Venere D., Nardi G.M., Lacarbonara V., Laforgia A., Stefanachi G., Corsalini M., Grassi F.R., Rapone B., PETTINI F. (2017). Early Mandibular Canine-Lateral Incisor Transposition: Case Report. Oral Implantol..

[45] Hu H., Chen J., Guo J., Li F., Liu Z., He S., Zou S. (2012). Distalization of the Mandibular Dentition of an Adult with a Skeletal Class III Malocclusion. Am. J. Orthod. Dentofac. Orthop..

[46] Cirulli N., Ballini A., Cantore S., Farronato D., Inchingolo F., Dipalma G., Gatto M.R., Alessandri Bonetti G. (2015). Mixed dentition space analysis of a southern italian population: New regression equations for unerupted teeth. J. Biol. Regul. Homeost. Agents.

[47] El-Bialy T. (2020). The Use of High Frequency Vibration and Clear Aligners in Management of an Adult Patient with Class III Skeletal Malocclusion with Open Bite and Severe Bimaxillary Protrusion: Case Report. Dent. J..

[48] Coloccia G., Inchingolo A.D., Inchingolo A.M., Malcangi G., Montenegro V., Patano A., Marinelli G., Laudadio C., Limongelli L., Di Venere D. (2021). Effectiveness of Dental and Maxillary Transverse Changes in Tooth-Borne, Bone-Borne, and Hybrid Palatal Expansion through Cone-Beam Tomography: A Systematic Review of the Literature. Medicina.

[49] Simon M., Keilig L., Schwarze J., Jung B.A., Bourauel C. (2014). Forces and Moments Generated by Removable Thermoplastic Aligners: Incisor Torque, Premolar Derotation, and Molar Distalization. Am. J. Orthod. Dentofac. Orthop..

[50] Dianiskova S., Rongo R., Buono R., Franchi L., Michelotti A., D’Antò V. (2022). Treatment of Mild Class II Malocclusion in Growing Patients with Clear Aligners versus Fixed Multibracket Therapy: A Retrospective Study. Orthod. Craniofacial Res..

[51] Inchingolo A.D., Ceci S., Patano A., Inchingolo A.M., Montenegro V., Di Pede C., Malcangi G., Marinelli G., Coloccia G., Garibaldi M. (2022). Elastodontic Therapy of Hyperdivergent Class II Patients Using AMCOP^®^ Devices: A Retrospective Study. Appl. Sci..

[52] Maspero C., Cappella A., Dolci C., Cagetti M.G., Inchingolo F., Sforza C. (2022). Is Orthodontic Treatment with Microperforations Worth It? A Scoping Review. Children.

[53] Di Venere D., Rapone B., Corsalini M. (2020). Dental Trauma in the Anterior Sector: An Analysis of the Predisposing Factors in a Group of Orthodontic Patients. Clin. Ther..

[54] Ravera S., Castroflorio T., Garino F., Daher S., Cugliari G., Deregibus A. (2016). Maxillary Molar Distalization with Aligners in Adult Patients: A Multicenter Retrospective Study. Prog. Orthod..

[55] Patano A., Di Venere D., Ceci S., Berate P., Candrea S., Babtan A.-M., Azzollini D., Piras F., Curatoli L., Corriero A. (2021). Essential Oils Utility Implications in Symptomatic Burning Mouth Syndrome. Balneo PRM Res. J..

[56] Ceci S., Berate P., Candrea S., Babtan A.-M., Azzollini D., Piras F., Curatoli L., Corriero A., Patano A., Valente F. (2021). The Oral and Gut Microbiota: Beyond a Short Communication. Balneo PRM Res. J..

[57] Inchingolo A.D., Cazzolla A.P., Di Cosola M., Greco Lucchina A., Santacroce L., Charitos I.A., Topi S., Malcangi G., Hazballa D., Scarano A. (2021). The Integumentary System and Its Microbiota between Health and Disease. J. Biol. Regul. Homeost. Agents.

[58] Dimonte M., Inchingolo F., Minonne A., Arditi G., Dipalma G. (2004). Bone SPECT in Management of Mandibular Condyle Hyperplasia. Report of a Case and Review of Literature. Minerva Stomatol..

[59] Sangalli L., Laffranchi L. (2022). Skeletal Class III Malocclusion Treated with Clear Aligners and Remote Digital Monitoring during the COVID-19 Pandemic. A Case Report. Sci. Arch. Dent. Sci..

[60] Staderini E., Meuli S., Gallenzi P. (2019). Orthodontic Treatment of Class Three Malocclusion Using Clear Aligners: A Case Report. J. Oral Biol. Craniofacial Res..

[61] d’Apuzzo F., Minervini G., Grassia V., Rotolo R.P., Perillo L., Nucci L. (2021). Mandibular Coronoid Process Hypertrophy: Diagnosis and 20-Year Follow-Up with CBCT, MRI and EMG Evaluations. Appl. Sci..

[62] Malcangi G., Inchingolo A.D., Patano A., Coloccia G., Ceci S., Garibaldi M., Inchingolo A.M., Piras F., Cardarelli F., Settanni V. (2022). Impacted Central Incisors in the Upper Jaw in an Adolescent Patient: Orthodontic-Surgical Treatment—A Case Report. Appl. Sci..

